# Maturation process of regenerated tissues after single-stage simultaneous autologous particulated cartilage implantation and open wedge high tibial osteotomy for articular cartilage defects with medial osteoarthritis of bilateral knees: a case report

**DOI:** 10.1186/s12891-021-04368-5

**Published:** 2021-05-31

**Authors:** Yasushi Oshima, Norishige Iizawa, Shinro Takai, Tokifumi Majima

**Affiliations:** Department of Orthopaedic Surgery, Nippon Medical School, 1-1-5 Sendagi, Bunkyo-ku, Tokyo, 113-8603 Japan

**Keywords:** Case report, Knee osteoarthritis (knee OA), Open wedge high tibial osteotomy (OWHTO), Particulated cartilage implantation, T2 mapping magnetic resonance imaging

## Abstract

**Background:**

Open wedge high tibial osteotomy (OWHTO) is an effective treatment option for young and middle-aged active patients with medial unicompartmental knee osteoarthritis (OA). In addition, particulated cartilage implantation has been developed as a simple procedure for cartilage regeneration. Thus, to improve the OWHTO outcomes, a single-stage, simultaneous bilateral knee arthroscopic particulated cartilage implantation with OWHTO was performed.

**Case presentation:**

A 60-year-old male patient presented with severe bilateral knee pain, with grade 2 varus knee OA of the Kellgren–Lawrence classification. Primary arthroscopic evaluations based on the International Cartilage Repair Society grading system showed grade 3c articular cartilage defects of 1.5 cm in diameter at the center of the bilateral medial femoral condyles. Following bilateral OWHTO, the healthy cartilage tissue was harvested from the lateral wall of the unilateral femoral intercondylar notch and minced with the cartilage processor. Then, subchondral drillings and cartilage fragment implantations into the bilateral defects were performed arthroscopically. One year postsurgery, second-look arthroscopy findings revealed that the defects were filled with cartilage-like tissues. The maturation process of the regenerated tissues was confirmed with T2 mapping magnetic resonance imaging during the 3-year follow-up period. The patient could walk without a cane, and all Knee Injury and Osteoarthritis Outcome Score parameters were improved without any correction loss in 3 years.

**Conclusions:**

This is the first report to evaluate the maturation process of the implanted particulated cartilage tissue with T2 mapping magnetic resonance imaging for 3 years. The effect of chondral resurfacing procedure with OWHTO remains unclear; however, the implantation of arthroscopic particulated cartilage fragments is a single-stage and less-invasive procedure. This treatment could regenerate cartilage-like tissue in the present case. Therefore, this additional procedure could potentially improve the long-term outcomes of OWHTO.

## Background

Medial open wedge high tibial osteotomy (OWHTO) is regarded as an effective joint-preserving procedure for young and middle-aged active patients with medial unicompartmental knee osteoarthritis (OA) [1]. Chondral resurfacing procedures, such as subchondral drilling, microfracture, and abrasion arthroplasty, in combination with OWHTO have also been reported to promote cartilage regeneration [2]. More recently, an alternative method in the form of a single-stage particulated cartilage tissue implantation has gained attention for its simplicity, low cost, and potential to repair articular cartilage defects [3].

We present a 3-year follow-up case report of a simultaneous bilateral subchondral drilling, particulated cartilage implantation, and OWHTO for a middle-aged OA patient. The process of cartilage regeneration was observed under T2 mapping magnetic resonance imaging (MRI) during patient follow-up.

## Case presentation

**Fig. 1:**
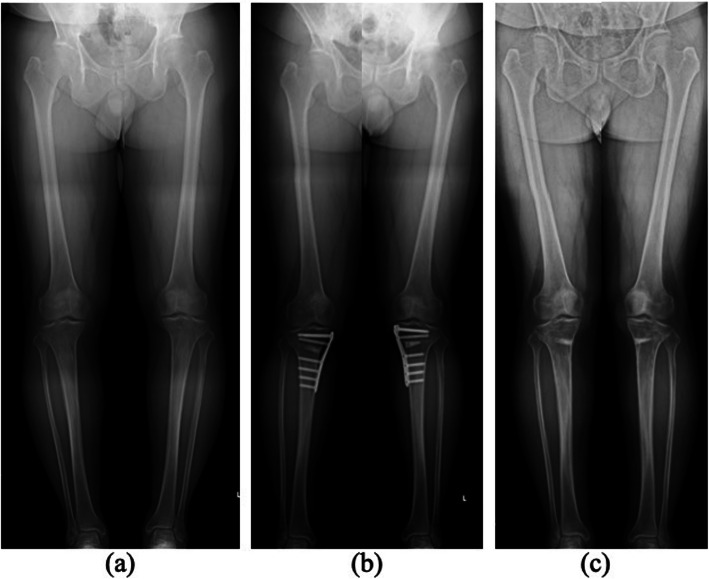
(**a**) Radiographical examination using a standing full weight-bearing lower extremity teleroentgenogram revealed varus knee angulation with a hip–knee–ankle (HKA) angle of − 2° on the right and − 4° on the left. (**b**) HKA angles were + 4° valgus on the right and + 5° valgus on the left 1 year after bilateral OWHTO. (**c**) Alignments of the lower extremities were preserved without correction loss 3 years after bilateral OWHTO and 2 years after the removal of fixations

A patient was a 60-year-old male who complained of severe bilateral knee pain. The patient was administrated nonsteroidal anti-inflammatory drugs, corticosteroids, and hyaluronic acid intraarticular injection for 2 years at the former clinic. However, the symptoms worsened, and he was referred to our hospital for the consideration of surgical treatment. The bilateral knee range of motion (ROM) was limited to be − 10° in extension and 130° in flexion, and he walked with the help of two canes. Radiographical examinations showed bilateral grade 2 knee OA in the Kellgren–Lawrence classification with a hip–knee–ankle (HKA) angle of − 2° on the right and − 4° on the left (Fig. 1a). Because of the severe pain and decrease in the activity of daily living, around the knee osteotomy surgery was performed at our hospital.

Arthroscopic evaluations revealed that the articular cartilage at the medial femoral condyle was softened and could be easily detached from the subchondral bone; the defects were classified as grade 3c according to the International Cartilage Repair Society (ICRS) grading system [4]. The degenerated cartilage was removed using a curette, and the defects measured 1.5 cm in diameter in both knees.

Bilateral OWHTO was performed with a correction angle of 8° on the right and 9° on the left, and the tibiae were fixed using beta-tricalcium phosphate spacers (Osferion 60, Olympus Terumo Biomaterials, Tokyo, Japan) and titanium locking plates (TriS medial HTO plate system, Olympus Terumo Biomaterials, Tokyo, Japan) (Fig. 1b) [5].

**Fig. 2:**
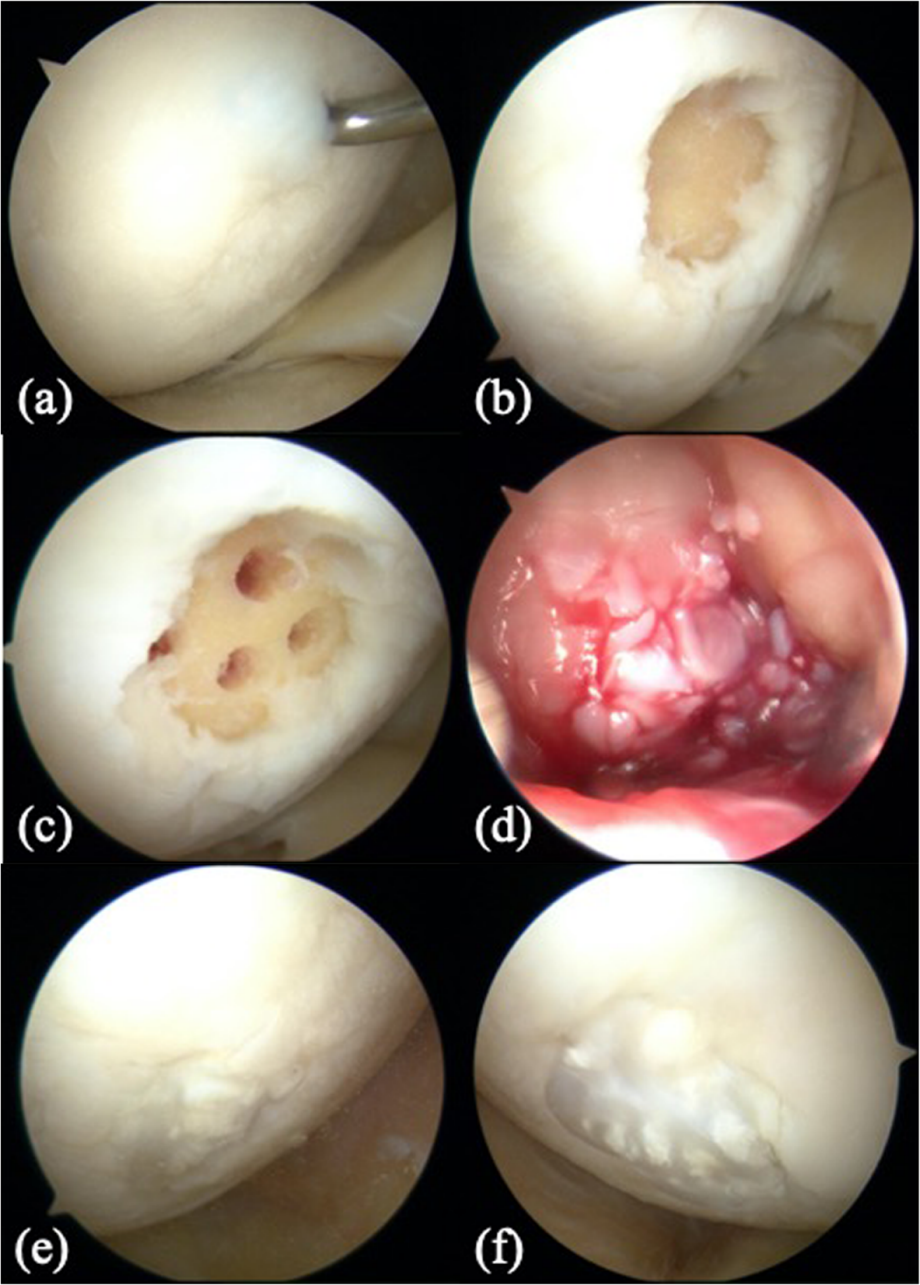
Arthroscopic findings and surgical procedures. (**a**) Softening of the articular cartilage at the medial femoral condyle was detected in the right knee. (**b**) Cartilage was debrided using a curette, and the defect was 1.5 cm in diameter. (**c**) Subchondral bone was drilled using a 1.8 mm Kirschner wire. (**d**) After dehydrating the knee joint, the particulated cartilage fragments were implanted into the defects and covered with fibrin glue. (**e, f**) Second-look arthroscopy evaluations demonstrated that the defects were filled with cartilage-like tissues on the right (**e**) and left (**f**)

Approximately 0.5 × 2 cm^2^-sized cartilage tissues were harvested from the lateral wall of the unilateral femoral intercondylar notch and minced with the cartilage processor (Reveille Cartilage Processor, Exactech Inc., Gainesville, FL, USA). The subchondral drilling technique was applied using a 1.8 mm Kirschner wire at a depth of 8 mm and a distance of 4 mm. After perfusate dehydration in the joint cavity, the particulated cartilage fragments were implanted and covered with fibrin glue arthroscopically (Beriplast, CLS Behring, King of Prussia, PA, USA) (Fig. 2).

The rehabilitation protocol was performed as per a previous report [3]. After surgery, the knee ROM exercise was initiated at up to 30° for 3 weeks, up to 60° for 6 weeks, and up to 90° for 8 weeks, and the full ROM exercise was permitted at 9 weeks. Partial weight-bearing (30%) was initiated at 3 weeks, 60% weight-bearing was initiated at 5 weeks, and full weight-bearing was allowed at 7 weeks.

**Fig. 3:**
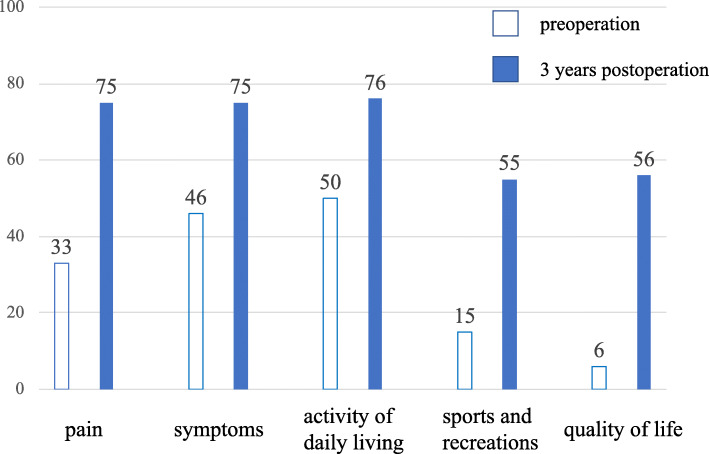
Knee Injury and Osteoarthritis Outcome Score (KOOS) variation. All parameters of KOOS were improved in 3 years

**Fig. 4:**
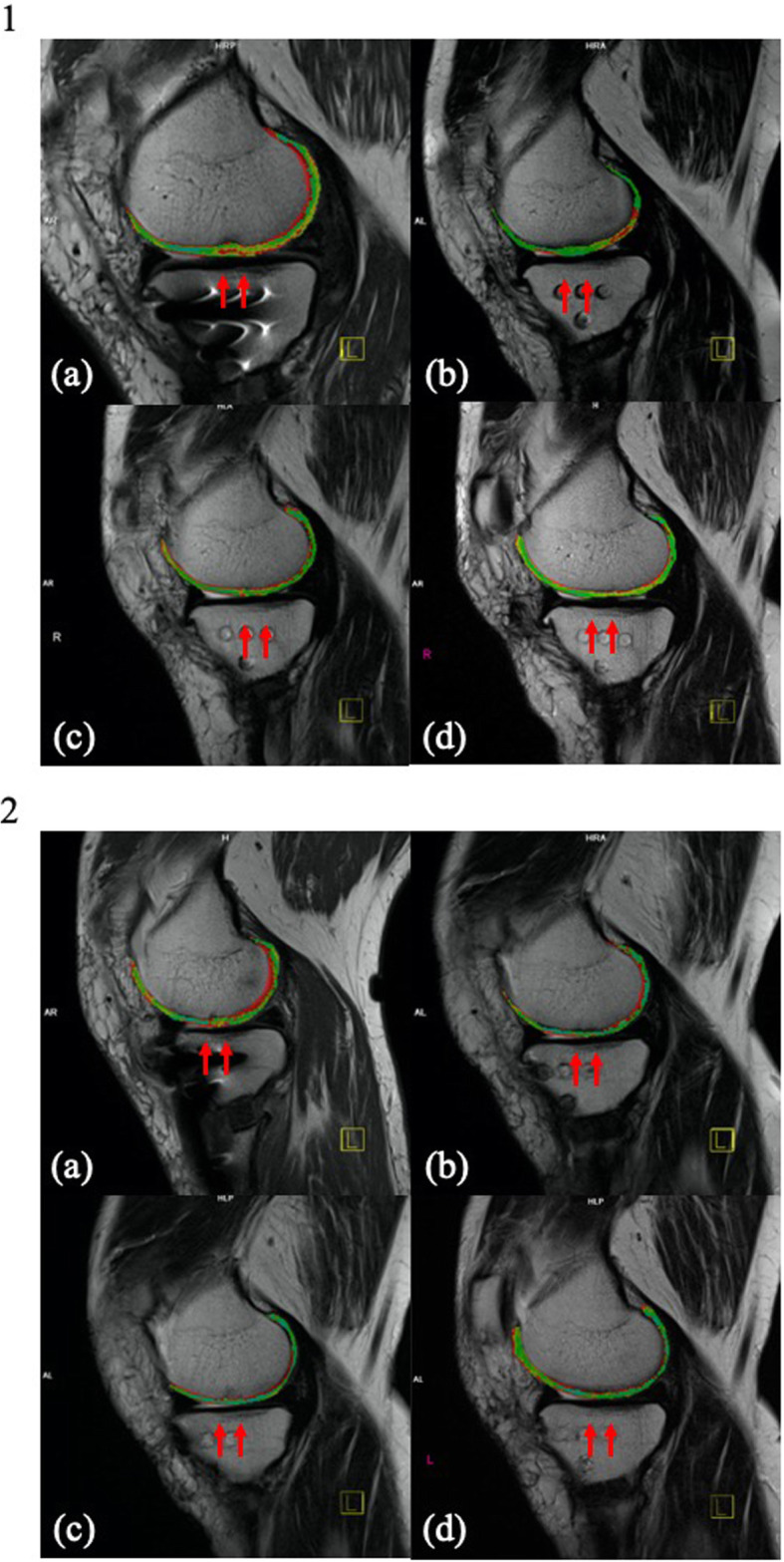
T2 mapping magnetic resonance examinations. (4–1) right knee, (4–2) left knee; the arrows illustrate the implanted sites. (4–1-**a**) On the right knee, the thicker regenerated tissues with abnormal signals were detected at the implanted site 6 months after the primary surgery. (**b**) The thickness of the regenerated tissues was still slightly greater than that of the surrounding cartilage; however, the abnormal signals were decreased 14 months after the primary surgery and 1 month after the removal of the fixations. (**c**) The thickness and signal of the regenerated tissues became similar to those of the surrounding cartilage 2 years after the primary surgery. (**d**) The regenerated tissues were preserved 3 years after the primary surgery. (4–2-**a**) On the left knee, the surface was not smooth, and abnormal signals were detected at the implanted site 3 months after the primary surgery. (**b**) The surface was still slightly irregular, and abnormal signals were detected 14 months after the primary surgery and 1 month after the removal of the fixations. (**c**) The thickness of the regenerated tissues became similar, and the signals were almost the same as those of the surrounding cartilage 2 years after the primary surgery. (**d**) The maturation of the regenerated tissues was observed 3 years after the primary surgery

Internal fixation removal with second-look arthroscopy was performed 13 months postoperatively. The defects were confirmed to be filled with regenerated tissues, which were mostly similar height level to the surrounding cartilage. The tissues integrated into the adjacent articular cartilage, and the demarcating borders were < 1 mm. Using a probe, the hardness of the surface at the regenerated tissues was found to be similar to the cartilage. Whitish spots were observed in the regenerated tissues, which were considered as the remaining original particulated cartilage matrix. Macroscopic appearance was not of an intact smooth surface; however, fibrillated change was undetected. From these findings, the regenerated tissues were diagnosed as grade II (nearly normal) with 9 out of 12 points in the ICRS cartilage repair assessment [4]. The donor site was also filled with fibrous tissue; however, the degenerated change was not observed at the surrounding articular surface. The bilateral knee ROM was increased to 0° in extension and 135° in flexion. All parameters of the Knee Injury and Osteoarthritis Outcome Score (KOOS) were improved without any correction loss in 3 years (Fig. 1c and 3). The maturation process of regenerated cartilage-like tissues was also observed under T2 mapping MRI (Fig. 4).

The patient felt a slight knee pain (75 in KOOS) during walking; however, it was improved from 33 in KOOS before the surgery, and he could walk without a cane for an hour. Thus, he felt satisfied with the surgical procedure using particulated cartilage implantation and OWHTO.

## Discussion and conclusion

The maturation processe of the regenerated tissues after single-stage bilateral autologous particulated cartilage implantation, subchondral drilling, and OWHTO was observed in T2 mapping MRI along with the improvement of clinical outcomes in a 3-year follow-up period.

The clinical results of OWHTO have been shown to be promising [6]. Moreover, the 10-year survival rate has been reported at 80–90% [1]. Furthermore, degenerated cartilage was previously confirmed to be covered with the repaired cartilage tissues only after OWHTO and without any additional chondral resurfacing techniques [7]. Thus, OA progression has been shown to decelerate in addition to knee pain relief after OWHTO, and the long-term clinical and radiological deterioration has been confirmed after OWHTO [8].

For the ICRS grade 3 defects, the regenerated tissues after OWHTO without any additional treatment have been confirmed as immature fibrocartilage tissues [9]. Therefore, in addition to the debridement of partially detached cartilage fragments, further treatments, such as the subchondral bone stimulation (drilling and microfracture) and perichondral or periosteal grafting, have been considered. The osteochondral graft and the cultured chondrocyte implantation have also been regarded as treatment options [4]. Nevertheless, a successful cartilage repair of chondral resurfacing procedures in OWHTO has been shown not to improve the clinical and radiological outcome [2]. Thus, the need and effect of these procedures remain unclear; however, several techniques have been suggested in combination with OWHTO to obtain cartilage regeneration and improved long-term outcomes.

In the present case, subchondral drilling and particulated cartilage tissue implantation in combination with OWHTO were performed for ICRS grade 3c defects with varus knee deformity. The use of minced cartilage for hyaline cartilage repair has been practiced in Germany since 1983. Its promising clinical outcomes have been recently reported with the migration of chondrocytes from cartilage chips and new extracellular matrix formation as well as the survivability of the chondrocytes after cartilage was minced with sharp instruments [10–12]. The particle size of minced cartilage was approximately 0.3–1.0 mm in diameter, and the operation was performed on < 4 cm^2^-sized cartilage defects. As the present cartilage processor system involves autologous tissue transplantation with single-stage surgery, it eliminates graft rejection. Moreover, it is cost-effective and only requires 10–15 min for the harvest, preparation, transplantation, and fixation of the cartilage tissues.

Using this procedure, we observed the maturation processe of the regenerated tissues near the surrounding native hyaline cartilage using T2 mapping MRI. T2 mapping is a quantitative technique that evaluates the interaction of water molecules and collagen network within the articular cartilage. This technique distinguishes the hyaline cartilage from the fibrocartilage [13, 14]. As defects were shown to be repaired with the matured hyaline-like cartilage, the long-term outcome can be improved using this transplantation method rather than repairing the defect with fibrocartilage tissue.

This present case involves some limitations that the histological and biomechanical evaluations of the regenerated tissues were not performed. However, the single-stage surgery and arthroscopic procedure were inexpensive and less invasive. Moreover, the maturation processe of the regenerated cartilage-like tissues with the improvement of clinical outcomes was noted. The benefit of chondral resurfacing procedure combined with OWHTO is, however, still unclear. Therefore, further evaluations are necessary. Nonetheless, particulated cartilage implantation with OWHTO could potentially repair medial OA in active patients for a long-term benefit.

## Data Availability

All data generated or analyzed during this study are included in this published article.

## Abbreviations

OWHTO: Open wedge high tibial osteotomy
OA: Osteoarthritis
MRI: Magnetic resonance imaging
ROM: Range of motion
HKA angle: Hip-knee-ankle angle
ICRS: International Cartilage Repair Society
KOOS: Knee Injury and Osteoarthritis Outcome Score
